# Spatial and Genomic Data to Characterize Endemic Typhoid Transmission

**DOI:** 10.1093/cid/ciab745

**Published:** 2021-08-31

**Authors:** Jillian S Gauld, Franziska Olgemoeller, Eva Heinz, Rose Nkhata, Sithembile Bilima, Alexander M Wailan, Neil Kennedy, Jane Mallewa, Melita A Gordon, Jonathan M Read, Robert S Heyderman, Nicholas R Thomson, Peter J Diggle, Nicholas A Feasey

**Affiliations:** Institute for Disease Modeling, Bill & Melinda Gates Foundation, Seattle, Washington, USA; Centre for Health Informatics, Computing, and Statistics, Lancaster University, Lancaster, United Kingdom; Department of Clinical Sciences, Liverpool School of Tropical Medicine, Liverpool, United Kingdom; Malawi -Liverpool Wellcome Programme, Blantyre, Malawi; Department of Clinical Sciences, Liverpool School of Tropical Medicine, Liverpool, United Kingdom; Department of Vector Biology, Liverpool School of Tropical Medicine, Liverpool, United Kingdom; Malawi -Liverpool Wellcome Programme, Blantyre, Malawi; Malawi -Liverpool Wellcome Programme, Blantyre, Malawi; Wellcome Sanger Institute, Cambridge, United Kingdom; Department of Paediatrics, University of Malawi the College of Medicine, Blantyre, Malawi; School of Medicine, Dentistry and Biomedical Sciences, Queen’s University Belfast, Belfast, United Kingdom; Adult Medicine, University of Malawi the College of Medicine, Blantyre, Malawi; Malawi -Liverpool Wellcome Programme, Blantyre, Malawi; Institute of Infection, Veterinary and Ecological Sciences, The University of Liverpool, Liverpool, United Kingdom; Adult Medicine, University of Malawi the College of Medicine, Blantyre, Malawi; Centre for Health Informatics, Computing, and Statistics, Lancaster University, Lancaster, United Kingdom; Division of Infection and Immunity, University College London, London, United Kingdom; Wellcome Sanger Institute, Cambridge, United Kingdom; Department of Pathogen Molecular Biology, London School of Hygiene and Tropical Medicine, London, United Kingdom; Centre for Health Informatics, Computing, and Statistics, Lancaster University, Lancaster, United Kingdom; Department of Clinical Sciences, Liverpool School of Tropical Medicine, Liverpool, United Kingdom; Malawi -Liverpool Wellcome Programme, Blantyre, Malawi

**Keywords:** *Salmonella* typhi, typhoid fever, genomics, spatial patterns, environmental transmission

## Abstract

**Background:**

Diverse environmental exposures and risk factors have been implicated in the transmission of *Salmonella* Typhi, but the dominant transmission pathways through the environment to susceptible humans remain unknown. Here, we use spatial, bacterial genomic, and hydrological data to refine our view of typhoid transmission in an endemic setting.

**Methods:**

A total of 546 patients presenting to Queen Elizabeth Central Hospital in Blantyre, Malawi, with blood culture–confirmed typhoid fever between April 2015 and January 2017 were recruited to a cohort study. The households of a subset of these patients were geolocated, and 256 *S.* Typhi isolates were whole-genome sequenced. Pairwise single-nucleotide variant distances were incorporated into a geostatistical modeling framework using multidimensional scaling.

**Results:**

Typhoid fever was not evenly distributed across Blantyre, with estimated minimum incidence ranging across the city from <15 to >100 cases per 100 000 population per year. Pairwise single-nucleotide variant distance and physical household distances were significantly correlated (*P* = .001). We evaluated the ability of river catchment to explain the spatial patterns of genomics observed, finding that it significantly improved the fit of the model (*P* = .003). We also found spatial correlation at a smaller spatial scale, of households living <192 m apart.

**Conclusions:**

These findings reinforce the emerging view that hydrological systems play a key role in the transmission of typhoid fever. By combining genomic and spatial data, we show how multifaceted data can be used to identify high incidence areas, explain the connections between them, and inform targeted environmental surveillance, all of which will be critical to shape local and regional typhoid control strategies.

## Teaser

This study identified significant spatial heterogeneity of multidrug-resistant typhoid fever across Blantyre, Malawi, where the febrile disease has been endemic since 2011. Hydrological catchments were predictive of genomic patterns observed, highlighting the role of environmental systems in ongoing transmission.

Water and sanitation improvements in the early 1900s led to effective elimination of typhoid fever in most high-income countries, without widespread use of either antibiotics or vaccines. However, typhoid remains a major cause of disease and death in low- and middle-income countries, with an estimated 11 million cases occurring annually [1]. In March 2018, the typhoid conjugate vaccine was recommended by the World Health Organization for control of typhoid, providing momentum for global initiatives to combat this disease [2]. Although this vaccine offers a high level of clinical protection [3], it does not completely prevent shedding of the disease [4]. Furthermore, modeling studies have shown that elimination using a vaccine alone is unlikely [5]. Multifaceted initiatives combining effective vaccines and detailed epidemiological surveillance, with water, sanitation, and hygiene (WASH) interventions must therefore be developed [6].

Although provision of clean water and sanitation is a global priority and a Sustainable Development Goal, the pace of these changes has not aligned with typhoid conjugate vaccine rollout in typhoid-endemic countries in Asia and Africa [7]. Targeted WASH strategies may support typhoid elimination on a more rapid timeline; however, targeting interventions is challenging, because typhoid transmission pathways do not appear to be consistent across locations [8, 9].

Typhoid transmission can occur through 2 modes. First, “short-cycle” transmission is characterized by food or water contamination in close proximity. This transmission pathway is frequently linked to food handlers [10] and transmission within the household [11].

Next, “long-cycle” transmission occurs by contamination of and contact with the external environment [12]. Although a human-restricted pathogen, little is known about the behavior of *Salmonella enterica* serovar Typhi in the environment between fecal excretion by one host and consumption by the next. Reservoirs for *S.* Typhi may exist in the environment, including amebas, allowing for extended survival outside the body [13]. Environmental mediators vary by location: the contamination of drinking water through stone taps in Kathmandu, Nepal, has been hypothesized as a major driver of transmission in this setting [8], while in Santiago, Chile, produce contaminated with sewage was the key driver of transmission; drinking water was not considered a risk factor in this setting [9]. In Blantyre, Malawi, the use of river water for cooking and cleaning was identified as a risk factor for typhoid fever [14], providing further evidence for diverse environmental exposures to this pathogen.

Long-cycle transmission pathways are technically difficult to identify, partly because *S.* Typhi is challenging to isolate from environmental matrices [15]. In the absence of effective environmental surveillance for typhoid, we hypothesized that geolocation of cases and sequencing of their isolates will help elucidate our understanding of long-cycle transmission pathways and environmental mediators of the disease.

In the current study, we investigated typhoid fever in Blantyre, where a rapid emergence of multidrug-resistant *S*. Typhi occurred in 2011 [16]. We used a geostatistical modeling framework to combine genomic information from isolates with georeferenced hydrological, geographic, and demographic data. The current study’s cohort contained a nested case-control study, which previously found a complex network of potential risk factors of typhoid fever related to both availability of WASH and the use of river water for cooking and cleaning, as well as social exposures such as daycare attendance [14]. Therefore, we specifically aimed to further investigate role of hydrological systems as a driver of transmission in this typhoid-endemic setting.

## METHODS

### Setting and Case Ascertainment

Queen Elizabeth Central Hospital (QECH) provides free secondary healthcare to the Blantyre urban area and surrounding district, as well as tertiary care to the southern region of Malawi. The Malawi-Liverpool Wellcome Programme has been conducting sentinel surveillance of bloodstream infections at QECH since 1998 [17]. Patients living in Blantyre who had blood culture–confirmed typhoid fever diagnosed between April 2015 and January 2017 at QECH were included in a prospective observational cohort. Age, residential area, human immunodeficiency virus (HIV) status, inpatient versus outpatient treatment, clinical presentation, complications, and deaths were recorded from clinical case records and/or during patient interviews.

Geolocation and spatial analysis were restricted to patients residing in urban Blantyre. Residential location and location of any household water source used within 3 weeks before diagnosis were recorded using 2 methods. Starting in April 2015, the households of case patients who were enrolled in the nested case-control study of children [14] were geolocated by a field team during household visits, using Garmin Etrex 30 GPS devices. Beginning in August 2015, the electronic PArticipant Locator application (ePAL), a tablet-based geolocation system, [18] was used to remotely geolocate the households for the remainder of the cohort. Informed written consent was sought from adult participants and from the legal guardians of children.

### Ethics Statement

This study was approved by the University of Malawi, College of Medicine Research and Ethics Committee (no. P.08/14/1617), the Liverpool School of Tropical Medicine Research Ethics Committee (no. 14.042), and the Lancaster University Faculty of Health and Medicine Ethics Committee (no. FHMREC17014).

### Incidence Mapping

We estimated the minimum incidence of typhoid fever associated with presentation to QECH for the enumeration areas (EAs) of Blantyre. Population estimates were derived from a 2016 census of Blantyre and surrounding areas that divided the city into 275 EAs [19]. This census included population structure by age bands (<5, 5–14, ≥15 years) and the number of households. Numerous approaches to adjusting the incidence of typhoid fever have been proposed, based on healthcare utilization, the probability of a diagnostic being performed, and its sensitivity [20–22]; however, these were not available at the EA level. Thus, we adjusted only for the study recruitment rates as a proportion of all diagnosed cases. Finally, a healthcare utilization survey was administered to a subset of controls from the nested case-control study [14].

All statistical analyses were conducted using R statistical software, version 3.5.1 [23]. To estimate incidences across the city, a Poisson log-linear model with a spatial random effect was fitted using the PrevMap package (version 1.5.3) [24]. Rates were estimated for each EA and age band, and the estimated population size in each age band was included as an offset. The offset was weighted to account for the longer time period for geolocation of the case-control study participants versus the rest of the cohort. Covariate effects were explored, including distance from the centroid of each EA to QECH, average household size, population density, elevation at the centroid, and hydrological catchment (Supplementary Material 1). The statistical model is further described in Supplementary Material 2.

### Sequencing and Phylogenetic Analysis

To investigate the genetic patterns of *S*. Typhi in this study, we performed whole-genome sequencing of isolates of *S*. Typhi from the index patients, using Illumina HiSeq 10X machines generating 150–base pair paired-end reads. Reads were mapped against the high-quality reference genome of *S.* Typhi 1036491, isolated in Blantyre in 2012 (GCA_001367555.3). The pairwise single-nucleotide variant (SNV) matrix was generated from this alignment.

The phylogeny was based on the same alignment and reconstructed using iq-tree (version 1.6.5) [25] under the general time-reversible model, ascertainment correction, and with 1000 bootstrap replicates for branch support. The resulting tree was assessed for phylogenetic signal using TempEst software (version 1.5.1) [26], and root-to-tip correlation was calculated. The phylogenetic tree was reconstructed into a maximum-likelihood joint ancestral reconstruction tree. Detailed protocols for genomic analyses are found in Supplementary Material 3, with accession numbers listed in Supplementary Material Table 2.

### Spatiogenomic Modeling

First, we tested for correlation between SNV distance and spatial distance. Next, the pairwise distance matrix of all absolute differences of SNVs was mapped to 2 dimensions using principal coordinates (PCs) analysis (PCoA), a method of multidimensional scaling. PCoA takes as its input an *m*-by-*m* matrix of pairwise dissimilarities among *m* entities, in this case SNVs, and looks for a graphic representation of these *m* entities that best approximates this complete set of dissimilarities by straight-line distances in a low-dimensional space, typically 1 or 2 dimensional for ease of visualization. We refer to the resulting PC axes generated from this analysis as genetic scores. The values of these scores themselves are not directly interpretable; rather, the closer together a set of SNVs are on the graphic representation, the stronger their genetic relatedness. We used a linear model with a spatial random effect to predict genetic score across the city. Finally, based on results of a previous risk factor study from a subset of this cohort that pointed to river water as a potential exposure [14], we explored the ability of river catchment to predict genetic score.

## RESULTS

### Characteristics of Cohort

**Figure 1.**
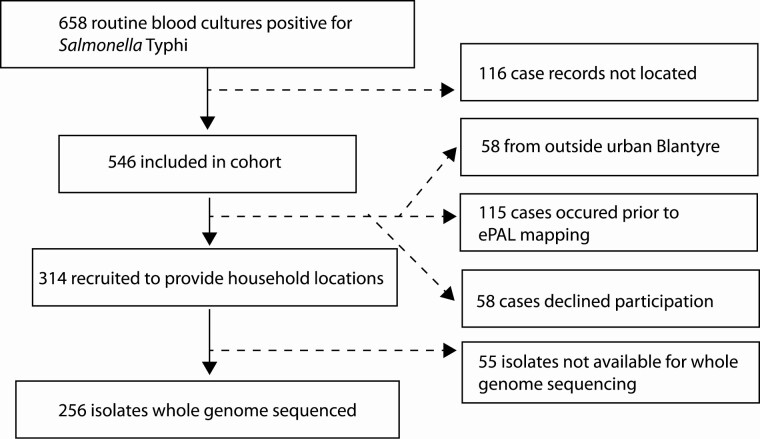
Consort chart outlining the process of recruiting individuals to the study, reasons for exclusion, and data availability of geographic and genomic data. Abbreviation: ePAL, the electronic PArticipant Locator application.

*S.* Typhi was isolated from 658 blood cultures between 28 March 2015 and 12 January 2017 (Figure 1), with an additional 2 isolates obtained from cerebrospinal fluid cultures. Of all isolates, 97% (641 of 660) were multidrug resistant, with resistance to ampicillin, chloramphenicol, and cotrimoxazole. Four patients were identified as having relapse or reinfection, with a second episode of *S.* Typhi bacteremia diagnosed 38–84 days after their first positive blood culture. A total of 546 illness episodes were included in the cohort study and are further referred to as cases; 484 case patients lived in the area of urban Blantyre and were therefore eligible for mapping, 314 consented to provide their household locations, and 256 isolates were available to be sequenced (Figure 1).

The characteristics of the cohort are summarized in Table 1 and presented in detail in Supplementary Material Table 1. The median age (IQR) was 11 (6–19) years, and the HIV seroprevalence was 10.7% (37 of 346 patients). Seventy-three percent of patients (391 of 542) were hospitalized, and hospital records were retrieved for 326. The case-fatality rate in the cohort was 1.5% (8 of 528).

**Table 1.** Characteristics of Recruited Patients With Typhoid Fever Presenting to Queen Elizabeth Central Hospital in Blantyre, Malawi

Characteristic or Outcome	Patients, No./Total (%)^a^
Age, median (range), y	11 (6–19)
Female sex	256/542 (47.2)
Positive malaria test result	7/533 (1.3)
Residence in urban Blantyre	484/542 (89)
Admitted to the hospital	391/542 (72.1)
Duration of hospital stay, median (IQR), d	4 (3–7)
Death	8/520 (1.5)

Abbreviation: IQR, interquartile range.

^a^Data represent no. (%) of patients unless otherwise specified.

### Incidence Mapping in Blantyre

**Figure 2.**
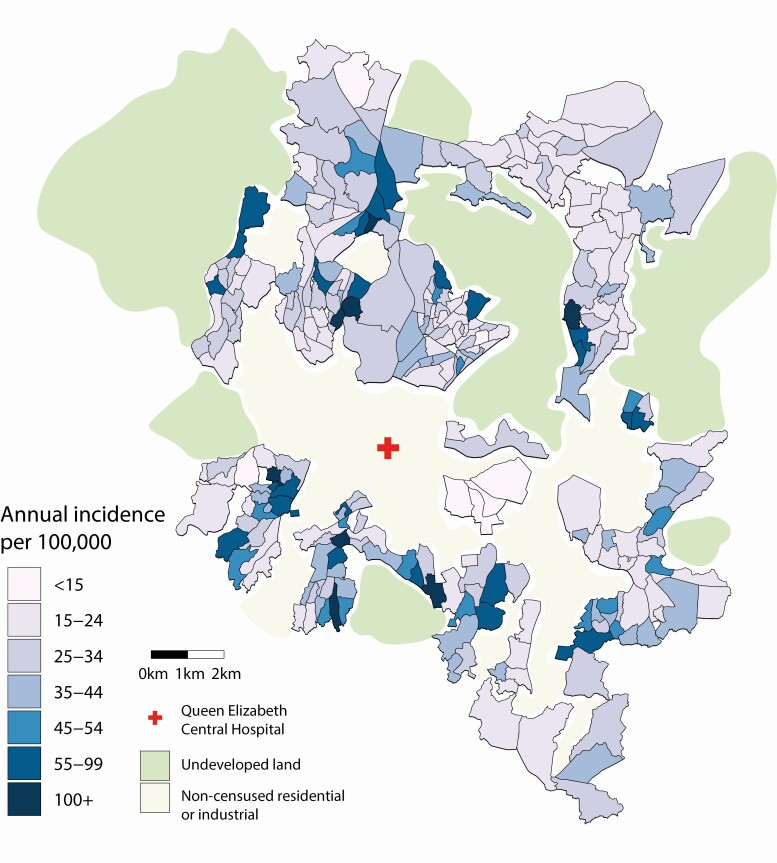
Estimated minimum annual incidence rate of typhoid fever for enumeration areas across the city of Blantyre, Malawi. The location of the recruiting hospital, Queen Elizabeth Central Hospital, is noted.

Of 658 case patients, 297 both consented to provide their household locations and lived within EA bounds and hence were included in the geostatistical incidence model. We adjusted resulting incidence rates for the number of patients who originated from urban Blantyre but were not included in mapping (n =115), declined participation (n = 58), or were lost to follow-up (n = 116). The estimated incidences in each EA are plotted in Figure 2. The model predicted the highest risk in the 5–14-year age band, followed by the ≥15-year age band (Table 2). Of the evaluated covariates, average household size was a significant predictor of incidence (Table 2), with smaller households indicative of a higher risk. Other tested covariates, including distance from hospital, did not significantly improve the model (Supplementary Material 2).

**Table 2.** Parameter Estimates for the Geostatistical Model of Typhoid Fever Incidence in Blantyre, Malawi

Parameter	Parameter Estimate (SE)	*P* Value
Intercept	−5.02 (0.60)	<.001
Average household size	−0.95 (0.14)	<.001
Age 5–14 y	1.08 (0.04)	<.001
Age <5 y	0.75 (0.04)	<.001
log(sigma2), spatially correlated variance	0.45 (0.11)	…
log(phi), range of spatial correlation	318 (0.17)	…
log(tau2), non-spatial variance	0.21 (0.23)	…

Model includes the significant tested covariate of average household size and is stratified by age band.

Abbreviation: SE, standard error.

The incidence across Blantyre was geographically heterogeneous, with an overall estimated minimum annual incidence rate of 36/100 000. Eight EAs were predicted to have an incidence rate >100/100 000, while 13 EAs had an incidence rate <15/100 000. Small-scale spatial correlation was identified in the region, with the range (phi) of this correlation estimated to be 318 m (Table 2). This indicates a practical range of spatial correlation (>5%) reaching approximately 950 m (Table 2).

Analysis of the healthcare utilization survey found no evidence that distance to hospital affects healthcare-seeking behavior for individuals included in this study. We found no significant difference between households that would or would not use QECH in the case of severe illness, based on the distance of their household to QECH (*P* = .28).

### Genomic Epidemiology

**Figure 3.**
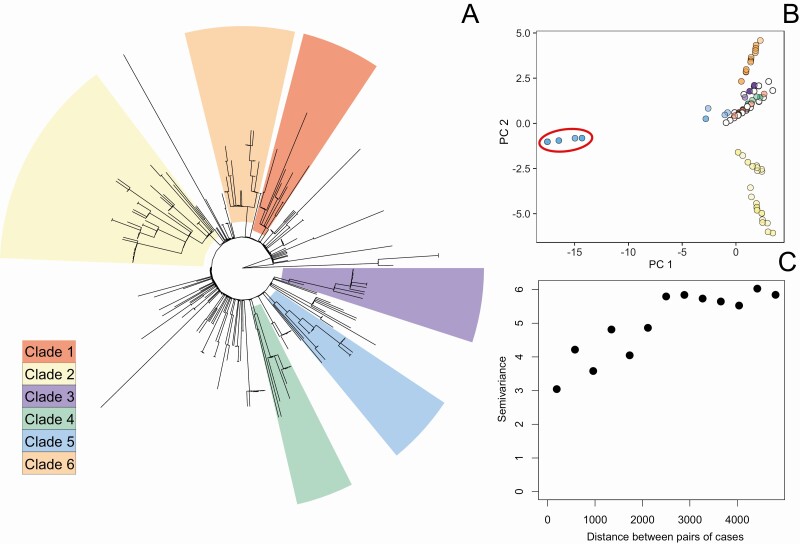
*A,* Joint ancestral state reconstruction tree based on whole-genome single-nucleotide variant (SNV) phylogenetic analysis for the sequenced isolates of *Salmonella* Typhi, showing the major clades of isolates determined using a root-to-tip directional approach. *B,* Further resolution of variation provided by decomposition of SNV matrix into the first 2 principal coordinates (PCs) of the multidimensional scale; colors of points represent membership in major clades, corresponding to the tree. *C,* Empirical semivariogram (proportional to 1 − spatial correlation as a function of distance) of PC2 of the SNV decomposition.

Isolates from 256 patients were whole-genome sequenced, and all belonged to the H58 haplotype, which has been identified globally, indicating sustained endemic transmission of this new strain in Blantyre. The isolates were highly clonal but could still be resolved into 6 phylogenetic clades (Supplementary Material 3 and Figure 3A). Root-to-tip correlation (0.07) indicated that the temporal signal was insufficient to enable temporal analysis (Supplementary Material 3).

The SNV distances between isolates and physical distances between households were significantly correlated (*P* = .001) (Supplementary Material 4). The first 2 PCs resulting from the PCoA accounted for 38% of the variation in the SNV matrix (Figure 3B). Scores along the primary axis (PC1) were similar for the majority of the cohort, with the exception of 11 isolates whose genetic score was approximately −15. This cluster of similar genetic scores suggests a distinct genetic group (highlighted in Figure 3B), contained within clade 5 in the tree (Figure 3A). However, these isolates did not differ spatially or temporally from the rest of the cohort (Supplementary Material 4). These isolates may indicate infections from a chronic carrier of *S.* Typhi or importations from another location with endemic H58.

PC1 showed no evidence of spatial correlation (Supplementary Material 4), so though this axis reflects an aspect of genetic relatedness, it cannot be explored using a geostatistical framework. However, genetic scores were more evenly distributed along PC2 (Figure 3B), indicating heterogeneity across this representation of the genetic signal. Furthermore, there appeared to be spatial correlation of these scores approaching 2500 m. This can be visualized with an empirical semivariogram (Figure 3C), which represents the variance of pairs of genetic score of isolates as a function of the distance between them. This observation was confirmed statistically (Supplementary Material 4). We therefore fitted the linear geostatistical model to PC2.

**Figure 4.**
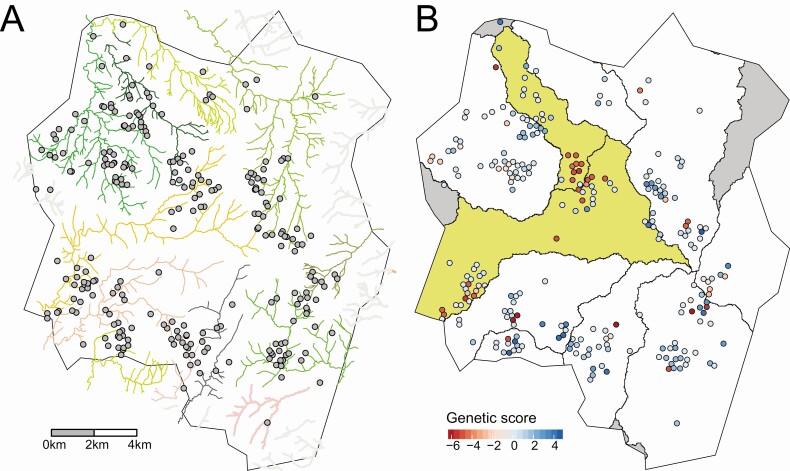
*A.* Major rivers of Blantyre with points indicating the approximate household locations of patients with typhoid fever. *B,* Approximate household locations are colored by genetic score, and river catchments are outlined using polygons. Catchments 2 and 8 are highlighted in yellow. Catchments not included in the analysis are in gray. Precise locations of households are masked by randomization.

Blantyre has a complex river network (Figure 4A), with 10 hydrological catchments including the cases identified (Figure 4B). Using hydrological river catchment as a categorical predictor in the linear model significantly improved the model’s fit to the genomic patterns observed, compared with an intercept-only model (log-likelihood −301.9 vs −289.4; *P* = .003). Parameter estimates indicated similar genetic scores for individuals in catchments 2 and 8 (Table 3), distinct from the other catchments. To confirm this observation, we conducted a contrast test to compare the mean coefficient values between catchments 2 and 8 versus the other river catchments. The difference was significantly different from zero, as evaluated using a *t* test (*P* < .001).

**Table 3.** Estimated Parameters for the Geostatistical Model of Genetic Scores, Representing the Genetic Heterogeneity in *Salmonella* Typhi Isolates.

Parameter	Estimate	Standard Error	*P* value
Catchment 1	0.092	0.34	.786
Catchment 2	−1.24	0.53	.020
Catchment 3	1.30	0.85	.128
Catchment 4	0.31	0.40	.443
Catchment 5	0.50	0.74	.500
Catchment 6	0.35	0.39	.363
Catchment 7	1.08	0.67	.105
Catchment 8	−1.09	0.33	.001
Catchment 9	0.81	0.48	.093
Catchment 10	0.64	0.45	.158
sigma^2^	4.116	1.106	-
phi	40.496	1.119	-
tau^2^	0.165	1.859	-

Hydrological catchment was included as a covariate in the model.

Abbreviations: CI, confidence interval; SE, standard error.

Estimates of spatial correlation of the geostatistical model highlighted the multiple scales of spatiogenetic clustering. The range parameter (phi) indicated that the practical range of spatial correlation (>5%) was approximately 192 m, indicating that the model’s spatial random effect was capturing short-distance spatial correlation. Although the city’s geographic range spans approximately 20 km, households in the cohort are clustered. 59% of the cohort has another cohort member within a distance of 192 m, and 13 households that were geolocated reported >1 case. We conducted a sensitivity analysis using geolocated water sources instead of household locations. Because the majority of individuals lived within close proximity of their water sources, the results were consistent with the findings using household location (Supplementary Material 4).

## Discussion

Geolocating cases as a part of routine surveillance has become increasingly common, allowing for spatially informed disease control, as for the targeting of polio vaccines [27], and the investigation of hot spots and transmission routes of Ebola [28] and severe acute respiratory syndrome coronavirus 2. Geospatial analyses for typhoid fever to date have revealed the spatially heterogeneous nature of the disease at both municipal and national scales [29, 30], but generalizable and epidemiologically relevant incidence covariates have yet to be identified [31]. In the current study, we demonstrated the utility of using statistical modeling to place multidimensional data sets in the context of a classic observational study, and in so doing we provide deeper insight into *S*. Typhi transmission hot spots. Initial mapping of the cohort revealed heterogeneity in incidence rates of typhoid across the city, and genomic analyses provided greater resolution of generalized endemic transmission of the disease, following a rapid emergence of a multidrug-resistant strain in 2011 [16].

Smaller average household size was significantly predictive of incidence rate in the current study. The increased incidence with decreasing household size, after accounting for population density and controlling for differential age distributions of EAs, may be a consequence of younger families living in greater socioeconomic precarity, but requires further investigation. Other studies have found increased risk in lower-elevation areas [32], which was not identified as a significant covariate in this study.

While reconstruction of phylogeny was successful in identifying discrete clades of *S*. Typhi, much granularity was lost by reducing the total genetic diversity present in 256 isolates to 6 categorical clades. Having excluded variable regions, we incorporated the full spectrum of remaining genetic variation by starting with all-against-all SNV distance followed by multidimensional scaling, which created a continuous variable for further analysis. Modeling the PCs extracted from the SNV matrix enabled us to view a continuous representation of genetic relatedness spatially, as well as test the predictive power of spatial covariates. A significant correlation between spatial and genetic distance was subsequently found, showing that typhoid fever patients living closer together were more likely to have *S.* Typhi isolates with closely related genomes. These findings support observations of generalized endemic transmission of typhoid fever, in contrast with a point source outbreak. Within this context, this study further demonstrated that in an endemic region like Malawi there is enough resolution, even within a single haplotype of *S.* Typhi, to infer likely hot spots of transmission.

Blantyre is delineated and divided by a complex river network, and, extrapolating from elevation maps, we can identify the numerous hydrological catchments across the city, areas within which any water or sewage will eventually converge to the same location. Adding river catchment to our spatial-genomic model resulted in a significantly improved fit. The importance of river catchment has also been proposed in Fiji, where heterogeneity of disease incidence between hydrological subcatchments was found [33]. The distinct differences in genomic patterns that appear to be delineated by hydrological catchments indicate that these hydrological zones may act as a unit of epidemiological mixing, which is plausible given the environmental component of long-cycle typhoid fever transmission. This is important when thinking about how potential herd effects across a city may be interpreted after a vaccination event, or how to target environmental surveillance to fully characterize a geographic region.

Small-scale spatial correlation also existed in the model after accounting for river catchment, which could reflect the short-cycle pathway of typhoid transmission and/or a common environmental exposure through attendance at school or daycare, identified as a risk factor in our nested case-control study [14]. Work to disentangle the underlying source of these exposures would help further elucidate these complex pathways.

There are limitations to the current analysis. Our single tested spatial covariate in the spatial-genomic analysis, grounded in previous work, was hydrological catchment. We currently lack predictors reflecting broader social interactions, such as school attendance or food sharing among households, which might also have contributed to the spatial clustering seen. We conducted a sensitivity test that revealed similar spatial-genomic patterns when using water source location versus household location; however, this does not sufficiently address the heterogeneity in potential exposures outside the home. Finally, we recruited only a subset of patients with typhoid, those seeking hospital care at QECH; analysis of bacterial genomes generated by active case finding or community-based surveillance that detects milder or subclinical cases would provide even deeper insight into *S.* Typhi transmission.

Currently, typhoid conjugate vaccines are being introduced in areas with known typhoid transmission; however, parallel WASH interventions are likely to be necessary for typhoid elimination, and identifying intervention points for these measures remains a challenge. In the current study, we paired spatial and genomic data to provide further evidence of the role that rivers may play in typhoid transmission. Previous work has indicated a risk with using river water for cooking and cleaning [14]. When paired with the current study, these findings may be used by targeted WASH-based public health messaging and interventions to control typhoid through a focus on ensuring all water, not just drinking water, is safe for household use. The next step will be to extend and validate these findings by direct detection of *S.* Typhi in these systems. Therefore, the development of methods for sensitive detection of *S*. Typhi in the environment will be critical to support the planning of public health interventions to interrupt transmission in the future.

## Supplementary Data

Supplementary materials are available at *Clinical Infectious Diseases* online. Consisting of data provided by the authors to benefit the reader, the posted materials are not copyedited and are the sole responsibility of the authors, so questions or comments should be addressed to the corresponding author.

ciab745_suppl_Supplementary_Materials_S1

ciab745_suppl_Supplementary_Materials_S2

ciab745_suppl_Supplementary_Materials_S3

ciab745_suppl_Supplementary_Materials_S4

ciab745_suppl_Supplementary_Table_S1

ciab745_suppl_Supplementary_Table_S2

## Notes

***Acknowledgments.*** We thank the staff and patients of Queen Elizabeth Central Hospital who supported and or consented to be recruited to this work, and we acknowledge expert informatics support from the Pathogen Informatics team at the Wellcome Sanger Institute.

***Financial support.*** This work was supported by the Bill & Melinda Gates Foundation (Investment OPP1128444) and the Wellcome Programme (grant 206454). The Institute for Disease Modeling is a research group within, and solely funded by, the Bill & Melinda Gates Foundation.

***Potential conflicts of interest.*** J. M. R. reports the following, all outside the submitted work: support from UK Research and Innovation (UKRI) Wellcome, paid to their institution; personal fees/consulting fees from Centra Technology; payment for teaching a course to the Ministry of Health, Saudi Arabia, paid to their institution; and service as a member of the Scientific Pandemic Influenza Group on Modelling, operational subgroup, Strategic Advisory Group of Experts on Immunization (SAGE), working to advise the UK government on the coronavirus pandemic (unpaid). All other authors report no potential conflicts. All authors have submitted the ICMJE Form for Disclosure of Potential Conflicts of Interest. Conflicts that the editors consider relevant to the content of the manuscript have been disclosed.
