# Supplementary material for: Spatial and Genomic Data to Characterize Endemic Typhoid Transmission
Source: Clin Infect Dis. 2021 Aug 31;74(11):1993–2000. doi: 10.1093/cid/ciab745 (PMC9187325; doi:10.1093/cid/ciab745)
Supplement: ciab745_suppl_Supplementary_Materials_S1 [file ciab745_suppl_supplementary_materials_s1.doc]

**Hydrological catchment definition using ArcGIS**

A digital elevation map was downloaded from USGS, two tiles spanning the Blantyre area were obtained, with data from the Shuttle Radar Thematic Mapper (SRTM), available in 1 arc-second resolution (approximately 30 meters), V3 [1].

All hydrological calculations used ArcGis Version 10.7 and ArcHydro tools 2.0. The DEM was reconditioned for consistency with a river map obtained from the Blantyre City Council. Flow direction was calculated, and estimated accumulation was visually compared to the known rivers, confirming agreement of the DEM with local maps (Figure S1). Pour-points were selected at the city limits, and along with the flow direction layer was used with Watershed tool to estimate hydrological catchments for major rivers.


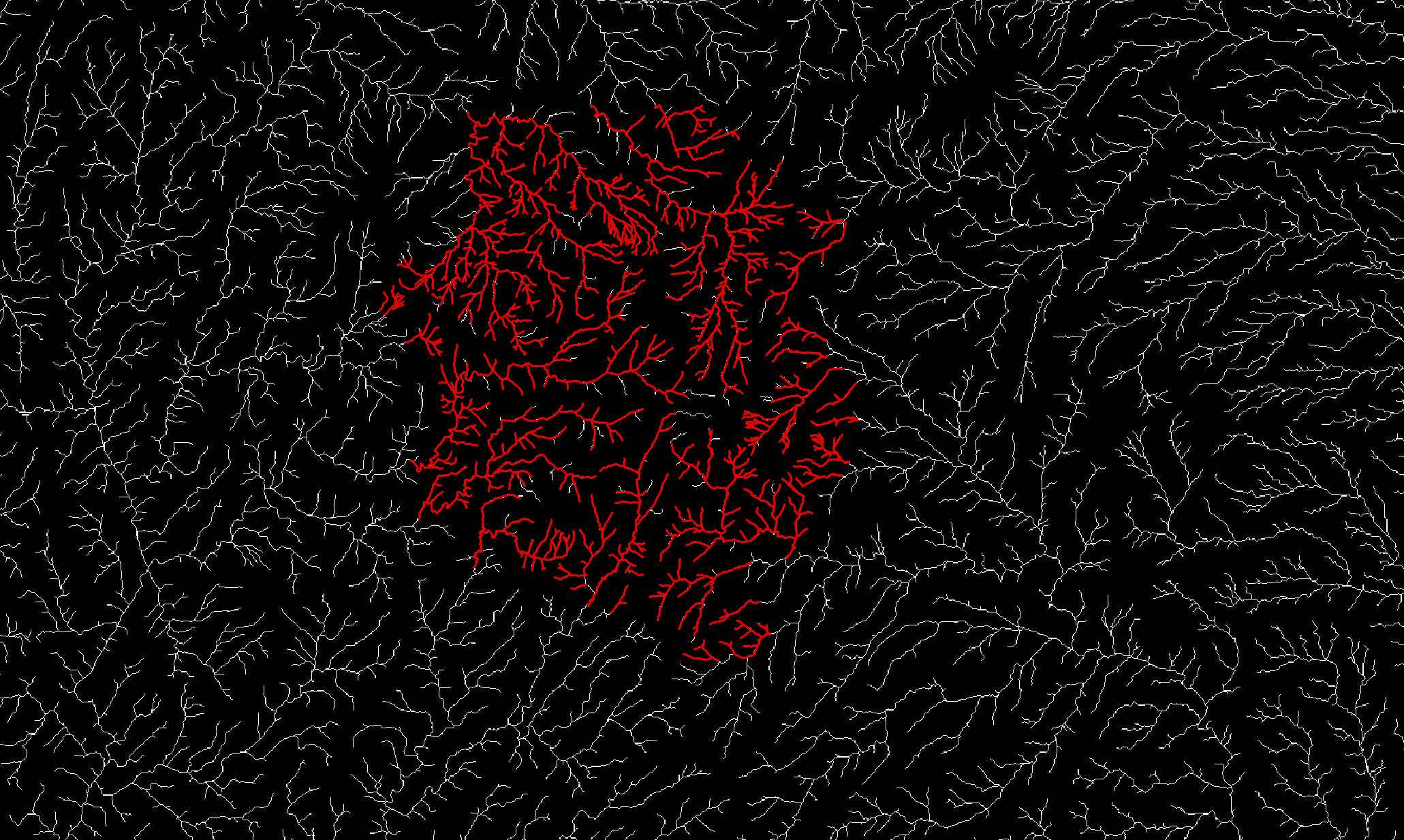


Figure S1. Map output from ArcMap showing estimated streams by flow accumulation (white), and known rivers (red).


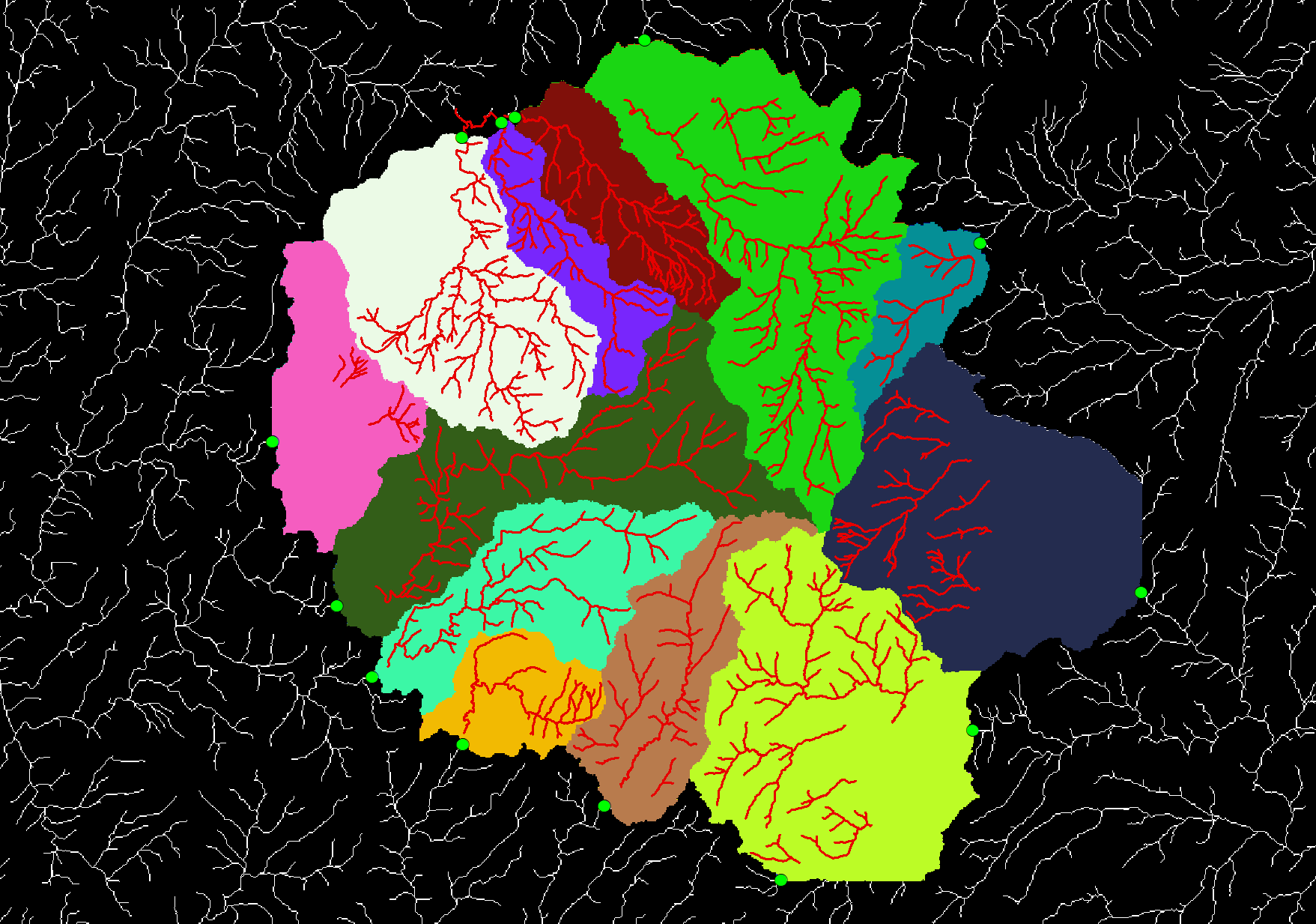


Figure S2. Pour points (green), and hydrological catchments in colored polygons.

1. USGS. SRTM Topography. SRTM Doc. 2009;
