# Supplementary material for: Spatial and Genomic Data to Characterize Endemic Typhoid Transmission
Source: Clin Infect Dis. 2021 Aug 31;74(11):1993–2000. doi: 10.1093/cid/ciab745 (PMC9187325; doi:10.1093/cid/ciab745)
Supplement: ciab745_suppl_Supplementary_Materials_S2 [file ciab745_suppl_supplementary_materials_s2.doc]

Supplementary Material 1: Incidence mapping

[**Non-spatial Poisson log-linear model** 2](#__RefHeading___Toc862455)

[**Assessing spatial dependence** 4](#__RefHeading___Toc862456)

[**Geostatistical model** 5](#__RefHeading___Toc862457)

# **Non-spatial Poisson log-linear model**

A Poisson log-linear model was used to model incidence across the city, initially with the assumption of no spatial dependence. We utilized available covariates for each enumeration area (EA): distance to QECH, elevation and river catchment at the centroid of the EA, and average household size and population density per square km across the enumeration area. For each enumeration area, we have age-stratified data of the population sizes in age bins of <5, 5-14, and 15+ years of age, and therefore can explore incidence rates in each enumeration area (*i*) and age band (*j*), where
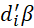
 represent enumeration area-specific predictors,
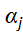
 are age-band specific intercepts, and
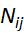
 are age-band and enumeration area-specific offsets.


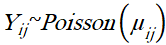


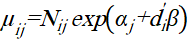


Results from the model are shown in Table S1. Estimated coefficients are relative to the 15+ age band. Average household size and age band were found to be significant predictors of incidence in the multivariate model. River catchment 6 was marginally predictive of an elevated incidence.

**Table S1. Estimated parameters from non-spatial Poisson log-linear model.**

| **Parameter** | **Estimate** | **Standard error** | **P value** |
| --- | --- | --- | --- |
| Intercept | -3.54 | 1.77 | 0.05 |
| Distance to QECH | -3.97E-05 | 6.52E-05 | 0.54 |
| Elevation | -0.0004 | 0.001 | 0.78 |
| Average household size | -1.05 | 0.26 | <0.001 |
| Density | -1.06E-05 | 7.28E-06 | 0.14 |
| Age 5-14 | 1.20 | 0.13 | <0.001 |
| Age <5 | 0.82 | 0.17 | <0.001 |
| Catchment 1 | 0.17 | 0.24 | 0.49 |
| Catchment 2 | -0.13 | 0.46 | 0.79 |
| Catchment 3 | 0.09 | 0.35 | 0.81 |
| Catchment 4 | 0.24 | 0.49 | 0.62 |
| Catchment 5 | -0.26 | 0.40 | 0.51 |
| Catchment 6 | 0.65 | 0.34 | 0.06 |
| Catchment 7 | -0.10 | 0.23 | 0.66 |
| Catchment 8 | -0.26 | 0.72 | 0.72 |
| Catchment 9 | -0.15 | 0.29 | 0.61 |
| Catchment 10 | 0.05 | 0.35 | 0.89 |

Covariates of average household size and age band were retained as the base model for further analyses. We explored the addition of any of the other four variables, but we found no significant (p<0.05) improvement in model fit with the addition of any of these variables (Table S2).Therefore, we used average household size as a predictor of incidence in each age band across the city for further analyses (Table S3).

**Table S2. Evaluation of added parameters**

| **Model** | **LL** | **P value** |
| --- | --- | --- |
| Base model | -596.66 | - |
| Base model + elevation | -596.20 | 0.33 |
| Base model + density | -595.68 | 0.16 |
| Base model + hospital distance | -596.11 | 0.29 |
| Base model + river catchment | -590.91 | 0.32 |

**Table S3. Summary of final model coefficients**

| **Parameter** | **Estimate** | **Standard error** | **P value** |
| --- | --- | --- | --- |
| Intercept | -4.98 | 0.94 | <0.001 |
| Average household size | 1.19 | 0.13 | <0.001 |
| Age 5-14 | 0.82 | 0.17 | <0.001 |
| Age <5 | -0.90 | 0.22 | <0.001 |

# **Assessing spatial dependence**

We explore whether spatial dependence of the residuals exists in the non-spatial model. We calculate the standardized Pearson residuals at the centroid of each enumeration area, *i*, by combining the expected counts
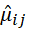
and observations *y*_ij_ for age band *j*:


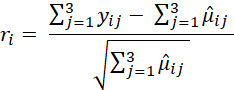


To test for spatial dependence, we randomly permutated the centroid locations *s=*500 times. We then constructed an empirical variogram for each permutation up to 10,000 meters, approximately ½ the linear dimensions of the study area. We calculated values for the empirical variogram as:


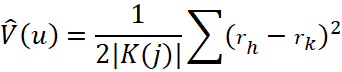


Where, for each value of *j,*  |*K(j)|* is the number of pairs in distance bin *j* and the summation is over all pairs *h* and *k* corresponding to pairs of locations whose distance apart falls within distance bin *j*. We calculated a 95% tolerance envelope of the variogram as the interval from the 13^th^ to the 487^th^ of the 500 ordered values of the corresponding variogram ordinates for each distance bin. The lower limit of the tolerance envelope lies substantially above 1 at all plotted distances, suggesting over-dispersion relative to the Poisson distribution. Also, the 95% tolerance envelope does not contain all points in the empirical variogram (Figure S1), suggesting the presence of some residual spatial correlation.

To test this formally, we define a test statistic to evaluate the variogram of the residuals from the final model against the null distribution generated by the randomly permutated centroid locations. This is generated for each permutation *i*, given in the equation below:


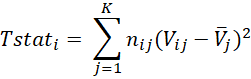


Where K is the number of variogram bins, V_ij_ is the calculated variogram ordinate in permutation *i* and bin *j.*
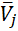
 is the weighted average of the variogram ordintes in bin *j* over N permutations:


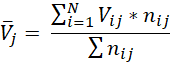


We then compare the test statistic of the variogram from our final model, *t*, with the calculated values from the permutated locations (Figure S2). The *p*-value of the test is


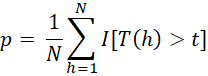


Where I[*a*>*b*]=1 if *a*>*b* and 0 otherwise. From this, we calculate the p-value to be 0.044. Based on this statistic and the above visualization, there appears to be significant evidence of spatial dependence in the data, meriting further analyses using an extended model that includes a spatial random effect.

**Figure S1. Empirical variogram of the residuals from the non-spatial generalized linear model, with the 95% tolerance envelope under the assumption of spatial randomness.**


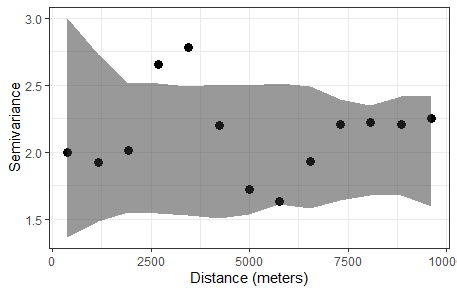


**Figure S2. Histogram of the calculated test statistics from 500 permutations, with the empirical test statistic shown in red.**

**
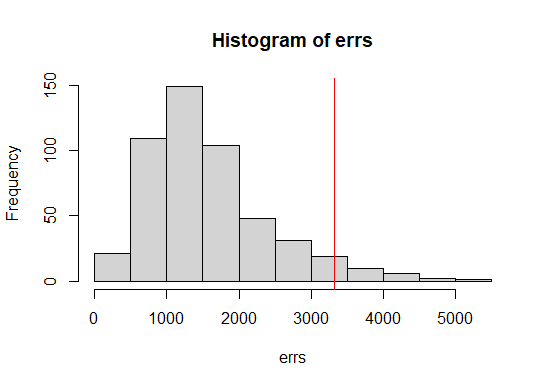
**

# **Geostatistical model**

Next, we extend our model to allow for over-dispersion and spatial dependence:


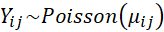


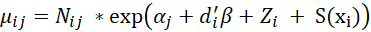


Where S(*x*) is a spatial random effect with a Matérn correlation function and kappa = 0.5 [1]. The model was fit using MCML, with 20,000 simulations, a burn-in of 10%, and a thinning parameter of 10. Initial values for the regression coefficients were taken from the fitted parameters of the non-spatial model, while the spatial covariance parameters (σ^2^, τ^2^, φ, representing the variance of S(*x*), variance of Z_i_, and the range of the spatial correlation, respectively) were estimated from a least-squares fit of the empirical variogram. MCML was repeated three more times, updating the initial values with estimates from the previous iteration. Diagnostics for the final iteration are shown for a randomly selected enumeration area in Table S3, with code used from PrevMap [2]. These diagnostics show little correlation between runs in thinned samples, visually apparent from the first and second columns, as well as a similar distribution of values in the first 900 and second 900 thinned samples, indicating stability in estimates over the iterations and convergence of the algorithm.

**Table S3. MCMC diagnostic plots for the geostatistical model indicating convergence.**

| **Age band** | **Trace of estimates by iteration** | **Autocorrelation of estimates between iterations** | **Cumulative distribution of estimates for first 900 and second 900 thinned samples** |
| --- | --- | --- | --- |
| **<5** | 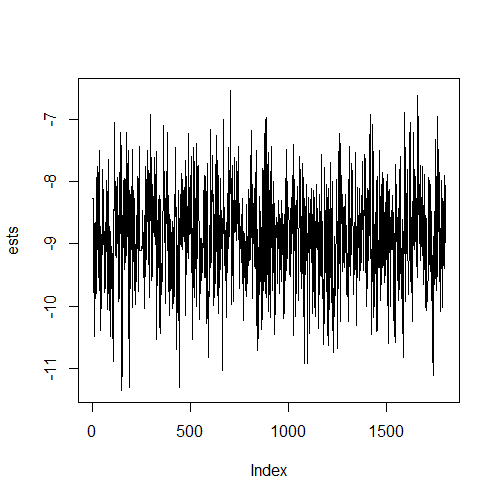 | 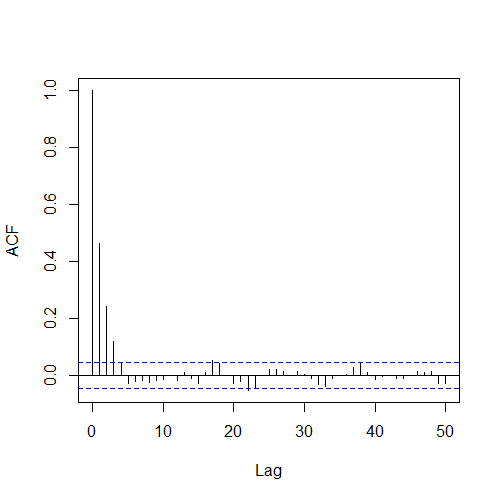 | 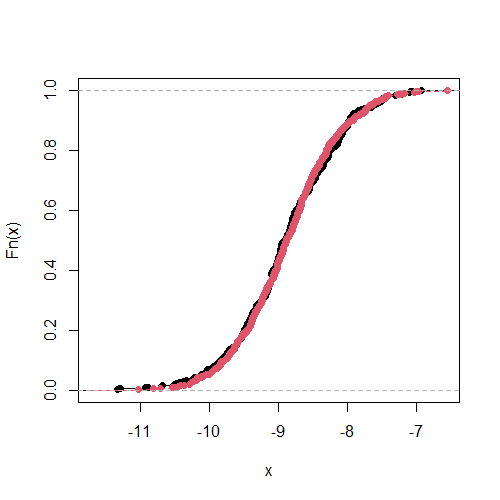 |
| **5-14** | 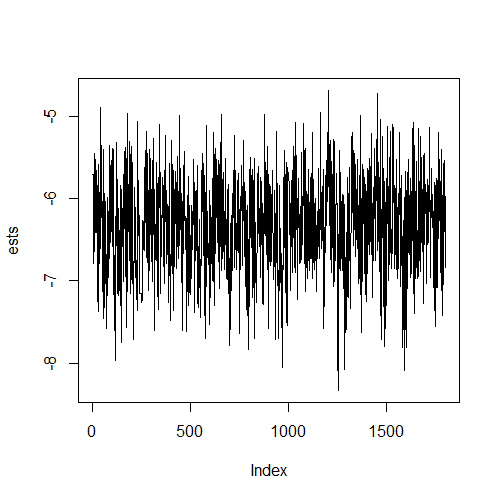 | 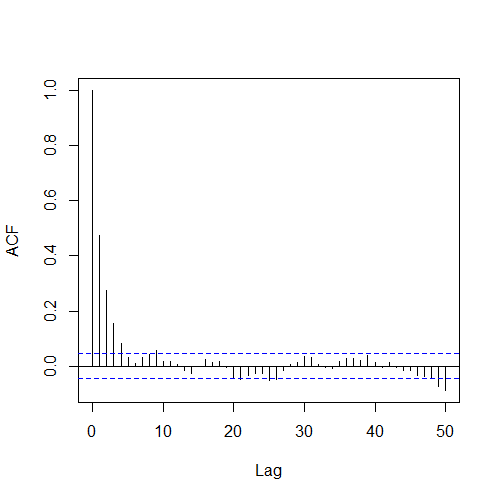 | 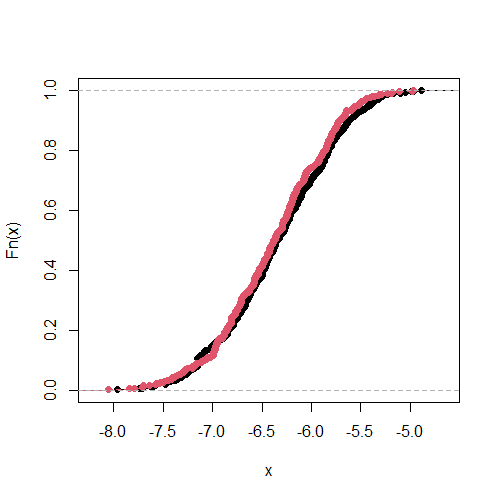 |
| **15+** | 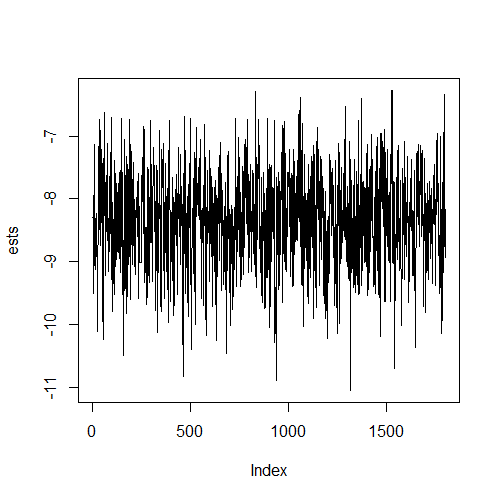 | 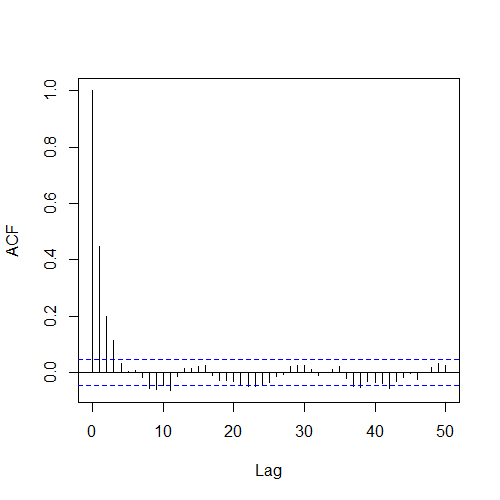 | 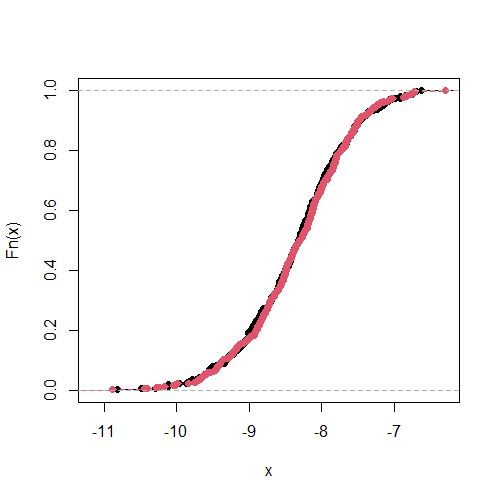 |

Estimated parameters for the non-spatial and spatial models are summarized in Table S4, and in the manuscript. Coefficient estimates appear similar to the nonspatial model estimates, though with reduced standard error (Table S4). The covariance parameters show that much of the variance in the model is captured in the nugget (τ^2^) relative to the sill (σ^2^), consistent with the over-dispersion observed in Figure S1, while the value phi indicates the practical range of spatial correlation (>5%) reaches approximately 1600 meters.

**Table S4. Parameter estimates for incidence model with and without spatial random effect.**

|  | Nonspatial model | | | Spatial model | | |
| --- | --- | --- | --- | --- | --- | --- |
| **Parameter** | **Estimate** | **Standard error** | **P value** | **Estimate** | **Standard error** | **P value** |
| Intercept | -4.98 | 0.94 | <0.001 | -5.02 | 0.60 | <0.001 |
| Average household size | 1.19 | 0.13 | <0.001 | -0.95 | 0.14 | <0.001 |
| Age 5-14 | 0.82 | 0.17 | <0.001 | 1.08 | 0.04 | <0.001 |
| Age <5 | -0.90 | 0.22 | <0.001 | 0.75 | 0.04 | <0.001 |
| σ^2^ | - | - | - | 0.45 | 0.11 | - |
| φ | - | - | - | 318 | 0.17 | - |
| τ^2^ | - | - | - | 0.21 | 0.23 | - |

Predictions of incidence at each centroid are calculated in PrevMap, again with 20,000 simulations, a burn-in of 10%, and a thinning parameter of 10. We additionally calculate the rates attributed to the covariate, as well as the rates attributed to the spatial signal. Our estimates can be separated into two components:


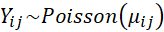


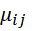
=
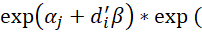
S(*x*))*
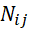


We want to estimate the components across all age bins *j* and for individual enumeration areas *i*. Using the additive properties of Poisson rates we can combine estimated
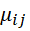
across all 3 age bands:


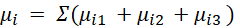


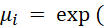
S(x))* [exp(
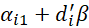
)*
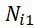
+ exp(
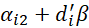
)*
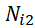
+ exp(
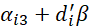
)*
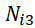
]

We calculate the contribution of the estimated covariates to the incidence directly, using estimated coefficients as


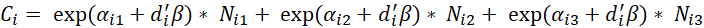
,

and the contribution of the spatial random effect as:

exp(S(*x*)) =
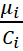


Each component is plotted in Figure S3. Some of the high incidence regions that appear in the model (Figure S3B) were attributed to the model covariate (Figure S3C), but others are not explained by measured covariates, and instead are captured by the spatial random effect (Figure S3A), indicating that there may be unmeasured processes contributing to these hot-spots.


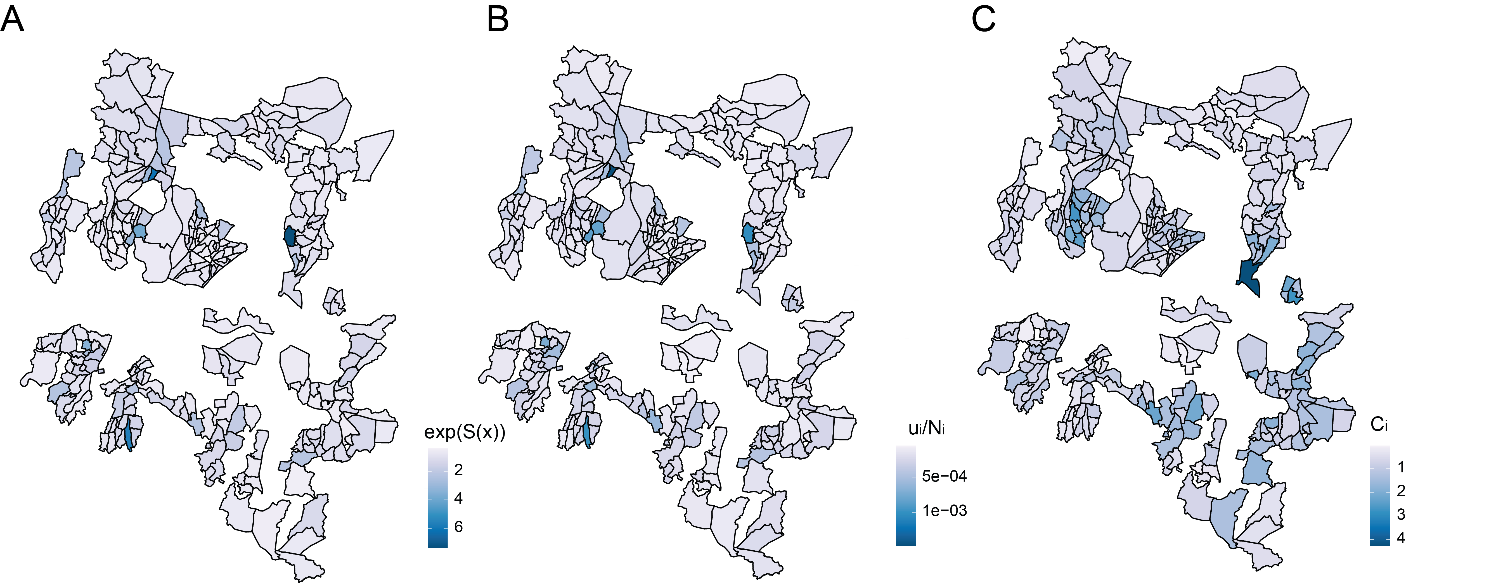


**Figure S3. Spatial (A) and covariate (C) attributed contributions to the model predicted incidence rate (B).**

1. Matérn, B.: Spatial Variation. 2nd Ed., Springer-Verlag, Berlin, Heidelberg, New York, London, Paris, Tokyo **1986**;

2. Giorgi E, Diggle PJ. **PrevMap** : An *R* Package for Prevalence Mapping. J Stat Softw **2017**;
