# Supplementary material for: Spatial and Genomic Data to Characterize Endemic Typhoid Transmission
Source: Clin Infect Dis. 2021 Aug 31;74(11):1993–2000. doi: 10.1093/cid/ciab745 (PMC9187325; doi:10.1093/cid/ciab745)
Supplement: ciab745_suppl_Supplementary_Materials_S3 [file ciab745_suppl_supplementary_materials_s3.doc]

# **Supplementary genomics methods**

*S*. Typhi from consenting participants were isolated, DNA extracted with the Qiagen Universal Biorobot® (Limburg, Netherlands) using Qiagen All-for-one® extraction kits, and subjected to whole genome sequencing on Illumina HiSeq2500 machines (Illumina, San Diego, CA, USA) generating 150 bp paired-end reads. Accession numbers for the read data are available in Supplementary Table 2. For the pan-genome analysis, annotated assemblies were produced using the pipeline described in [1]. For each sample, sequence reads were used to create multiple assemblies using VelvetOptimiser v2.2.5 (Velvet Optimiser: For automatically optimising the primary parameter options for the Velvet de novo sequence assembler. Gladman, S & Seemann, T, Victorian Bioinformatics Consortium, 2008. http://bioinformatics.net.au/software.velvetoptimiser.shtml) and Velvet v1.2[2]. An assembly improvement step was applied to the assembly with the best N50 and contigs were scaffolded using SSPACE [3] and sequence gaps filled using GapFiller[4]. Automated annotation was performed using PROKKA v1.5 [5] and a genus specific database from RefSeq[6].

All of the software developed by Pathogen Informatics at the WSI is freely available for download from GitHub (Pathogen Informatics, WSI, https://github.com/sanger-pathogens/vr-codebase; Bio-Assembly-Improvement: Improvement of genome assemblies by scaffolding and gapfilling, Pathogen Informatics, WSI, https://github.com/sanger-pathogens/assembly_improvement) under an open source license, GNU GPL 3. The improvement step of the pipeline is also available as a standalone Perl module from CPAN (http://search.cpan.org/~ajpage/). The core- and pan-genome were analyzed using roary [7] for gene-based comparisons.

**Single nucleotide variants (SNVs)**

Reads were mapped against the high-quality reference genome of *S.* Typhi 1036491 isolated in Blantyre, Malawi 2012 (GCA_001367555.3) using SMALT v0.7.4 (SMALT: A mapper for DNA sequencing reads, WSI, https://sourceforge.net/projects/smalt/) All bases were filtered to remove those with uncertainty in the base call. The bcftools variant quality score was required to be greater than 50 (quality < 50) and mapping quality greater than 30 (map_quality < 30). If not all reads gave the same base call, the allele frequency, as calculated by bcftools, was required to be either 0 for bases called the same as the reference, or 1 for bases called as a SNV (af1 < 0.95). The majority base call was required to be present in at least 75% of reads mapping at the base, (ratio < 0.75), and the minimum mapping depth required was 4 reads, at least two of which had to map to each strand (depth < 4, depth_strand < 2). Finally, strand_bias was required to be less than 0.001, map_bias less than 0.001 and tail_bias less than 0.001. If any of these filters were not met, the base was called as uncertain. An alignment was constructed by substituting the base call at each site (variant and non-variant) in the BCF file into the reference genome and any site called as uncertain was substituted with an N for each respective isolate.

**Phylogenetic analyses**

A pairwise SNV distance matrix of this alignment was generated selecting only sites containing ACGT (no gaps or Ns) using snp_sites[8], resulting in 436 informative sites for the pairwise comparison, used for further geo-spatial modelling. For phylogenetic analyses, isolate 53789 (ERR279153) was included in the mapping analysis as above and in further analyses, representing the related GenoTyphi type 4.1.1[9]. Recombinant sites and mobile elements were removed following analysis of the mapping-based alignment with gubbins v2.3.4 [10] as well as phage characterization using PHASTER [11]and manually curating the output; the coordinates of masked sites are given in Table S6. Informative sites were then extracted from this alignment using snp_sites [8]; only sites containing ACGT (no gaps or Ns) were used for the final analysis, resulting in 409 informative SNVs in the final alignment . For the phylogenetic analyses, the informative SNV alignment was used as input for iq-tree [12] for phylogenetic tree reconstruction under the general time-reversible (GTR) model, ascertainment (ASC) correction for a SNP-only alignment and under Gamma distribution (-m GTR+G+ASC), support was assessed using 1000 bootstrap replicates. The resulting tree was assessed for phylogenetic signal using tempest (v1.5.1;[13]) and the isolate collection days as recorded by QEH, however the root-to-tip correlation (0.07) indicated not enough temporal signal to allow a temporal analysis (supplement). The phylogenetic tree was rooted at isolate 53789 and reconstructed into a joint ancestral tree using pyjar (https://github.com/simonrharris/pyjar), which is a wrapper for the method described in [14], and rPinecone [15] was used to further group the isolates based on this tree, using 2 and 4 as relevant SNP cutoffs for minor and major clusters, respectively (figure 3A, main text). The outgroup isolate was removed from the tree visualization to improve viewability. Tree and metadata were visualized using ggplot (Book: Wickham, Hadley, Ggplot2 Elegant Graphics for Data Analysis.) and the ggtree package. [16] Pairwise tip-to-tip distances were calculated using the adephylo package for R with the command distTips [17] from the alignment before recalculation with pyjar.

**Table S6:** Coordinates of masked sites in reference genome GCA_001367555.3

| Start position | End position in |
| --- | --- |
| 1105746 | 1115242 |
| 1878360 | 1922985 |
| 2407793 | 2441813 |
| 2650078 | 2656238 |
| 3957201 | 3975207 |
| 4150847 | 4199187 |
| 4507080 | 4557892 |
| 4793916 | 4793950 |
| 1807617 | 1807636 |
| 87190 | 87232 |

1. Page AJ, De Silva N, Hunt M, et al. Robust high-throughput prokaryote de novo assembly and improvement pipeline for Illumina data. Microb Genomics **2016**;

2. Zerbino DR, Birney E. Velvet: Algorithms for de novo short read assembly using de Bruijn graphs. Genome Res **2008**; 18:821–829.

3. Boetzer M, Henkel C V., Jansen HJ, Butler D, Pirovano W. Scaffolding pre-assembled contigs using SSPACE. Bioinformatics **2011**;

4. Boetzer M, Pirovano W. Toward almost closed genomes with GapFiller. Genome Biol **2012**; 13.

5. Seemann T. Prokka: Rapid prokaryotic genome annotation. Bioinformatics **2014**; 30:2068–2069.

6. Pruitt KD, Tatusova T, Brown GR, Maglott DR. NCBI Reference Sequences (RefSeq): Current status, new features and genome annotation policy. Nucleic Acids Res **2012**;

7. Page AJ, Cummins CA, Hunt M, et al. Roary: Rapid large-scale prokaryote pan genome analysis. Bioinformatics **2015**;

8. Page AJ, Taylor B, Delaney AJ, et al. SNP-sites: rapid efficient extraction of SNPs from multi-FASTA alignments. Microb genomics **2016**; 2:e000056.

9. Wong VK, Baker S, Connor TR, et al. An extended genotyping framework for Salmonella enterica serovar Typhi, the cause of human typhoid. Nat Commun **2016**;

10. Croucher NJ, Page AJ, Connor TR, et al. Rapid phylogenetic analysis of large samples of recombinant bacterial whole genome sequences using Gubbins. Nucleic Acids Res **2015**; 43:e15.

11. Arndt D, Grant JR, Marcu A, et al. PHASTER: a better, faster version of the PHAST phage search tool. Nucleic Acids Res **2016**; 44:W16–W21.

12. Nguyen LT, Schmidt HA, Von Haeseler A, Minh BQ. IQ-TREE: A fast and effective stochastic algorithm for estimating maximum-likelihood phylogenies. Mol Biol Evol **2015**; 32:268–274.

13. Rambaut A, Lam TT, Carvalho LM, Pybus OG. Exploring the temporal structure of heterochronous sequences using TempEst (formerly Path-O-Gen). Virus Evol **2016**; 2:vew007.

14. Pupko T, Pe’er I, Shamir R, Graur D. A fast algorithm for joint reconstruction of ancestral amino acid sequences. Mol Biol Evol **2000**; 17:890–896.

15. Wailan AM, Coll F, Heinz E, et al. rPinecone: Define sub-lineages of a clonal expansion via a phylogenetic tree. Microb Genomics **2019**; 5:1–9.

16. Yu G, Lam TTY, Zhu H, Guan Y. Two methods for mapping and visualizing associated data on phylogeny using GGTree. Mol Biol Evol **2018**;

17. Jombart T, Balloux F, Dray S. adephylo: New tools for investigating the phylogenetic signal in biological traits. Bioinformatics **2010**;
