# Supplementary material for: Spatial and Genomic Data to Characterize Endemic Typhoid Transmission
Source: Clin Infect Dis. 2021 Aug 31;74(11):1993–2000. doi: 10.1093/cid/ciab745 (PMC9187325; doi:10.1093/cid/ciab745)
Supplement: ciab745_suppl_Supplementary_Materials_S4 [file ciab745_suppl_supplementary_materials_s4.doc]

Supplementary Material 4: Spatio-genetic model

[**Motivation for spatial analysis: Correlation** 2](#__RefHeading___Toc28336547)

[**Exploration of multidimensional scales: PC 1** 3](#__RefHeading___Toc28336548)

[**Exploration of multidimensional scales: PC 2** 5](#__RefHeading___Toc28336549)

[**Geostatistical modeling process** 6](#__RefHeading___Toc28336550)

[**Sensitivity analyses with household water source location** 8](#__RefHeading___Toc28336551)

# **Motivation for spatial analysis: Correlation**

Though it is commonly assumed that epidemiologically-linked individuals tend to have genetically related isolates, due to differences in transmission patterns between diseases, it is less established that spatially-close individuals are genetically linked. Therefore prior to geostatistical modeling of genetic data, we explored the correlation between spatial and genetic distances in our dataset.

The SNP data is represented as an *n*x*n* matrix of genetic distances. Using the household location of the patients, we then generated spatial distances between all patients. Next, the correlation between physical distance and SNP distance for all combinations of isolates was calculated, resulting in a value of 0.071.

In order to test the significance of this value, we randomly permuted the location labels of the individuals included in the genetic distance matrix, and calculated the correlation between SNP distance and physical distance. This process was repeated 1000 times.

We then compared our empirical test statistic, *t*, with those generated from the randomized values, C(*h*), using the calculated p value:


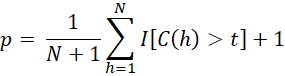


Where N is the number of permutations, I[*a*>*b*]=1 if *a*>*b* and 0 otherwise. The distribution of the permuted test statistics is shown in Figure 1, with the empirical test statistic shown in red. The resulting p value is 0.001, indicating that there is evidence of spatial-genetic correlation in our dataset.

**Figure S1. Histogram of calculated test statistics from 1000 permutations, with the empirical test statistic shown in red.**

# **Exploration of multidimensional scales: PC 1**

The semivariogram in Figure S2 does not suggest that PC 1 of the multidimensional scale has any spatial correlation up to 5 km.

**Figure S2. Semivariogram of genetic score for PC 1.**

Regardless, there were 11 individuals with a genetic score of approximately -30 on PC 1 of our multidimensional scale of the pairwise SNP distance matrix. Available covariates to investigate these individuals were age, time of infection, and household location (Figure S3). No significant difference in average age exists between these individuals and the rest of the cohort (13.8 vs. 15.7, p=0.67).

**Figure S3. Spatial distribution of cases (left), and cumulative proportion of cases over the study period (right), with the investigated individuals highlighted in red.**

To evaluate spatial clustering of these individuals versus the rest of the cohort, we generated K-functions across the study region up to 5000 meters, and used a statistical test for point process clustering [1]. The test statistic is evaluated from the difference between K-functions evaluated at distances (Figure S4), divided by the standard error of these differences, across the study region:


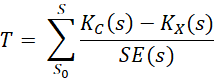


Randomly permutating the labels for K_c_ and K_s_, and repeating 500 times to create a null distribution, we generate a p-value of 0.072 indicating weak evidence of spatial clustering compared to the rest of the cohort. Given the small number of individuals in the evaluated group, there is little evidence to contribute to further conclusions regarding these individuals.

**Figure S4. K(s) at evaluated distances for the entire cohort (black) and evaluated individuals (red), and the difference in K-function estimate for the evaluated individuals compared to the rest of the cohort with dashed lines indicating 2+/- the standard error.**

A similar approach was used to evaluate clustering the study period, with position in space (in two dimensions) replaced by one-dimensional position in time. The p-value for clustering over time was calculated as 0.5. Therefore, although this group shows distinct differences in genetic scores of PC1 in relation to the rest of the cohort, these individuals do not appear to be related in time or space, and do not show unique characteristics regarding age at infection.

# **Exploration of multidimensional scales: PC 2**

The semivariogram of PC 2 shows visual evidence of spatial correlation (Manuscript Figure 2C), therefore we conducted a statistical test to observe whether this pattern is significant. To test for spatial dependence, we randomly permuted household locations of each isolate 500 times. We then constructed a semivariogram for each permutation up to 5,000 meters, approximately 1/4 the range of the study area. We calculated 95% tolerance envelope of the semivariogram as the interval from the 13^th^ to the 487^th^ of the 500 ordered values of the corresponding semivariogram ordinates for each distance bin. The 95% tolerance envelope does not contain all points in the empirical semivariogram (Figure S5).


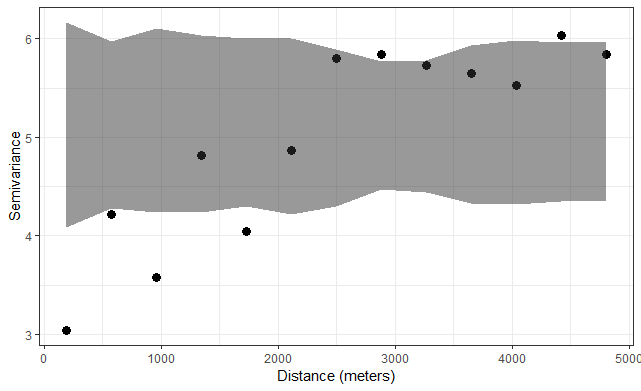


**Figure S5. Empirical semivariogram of PC 2 (points), with 95% tolerance envelope in shaded band.**

We further define a test statistic to evaluate the semivariogram of the residuals from the final model against the null distribution generated by the randomly permutated household locations. This is generated for each permutation *i*, given in the equation below:


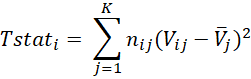


Where K is the number of semivariogram bins, *V_ij_* is the calculated semivariogram ordinate in permutation *i* and bin *j.*
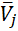
 is the weighted average of the semivariogram ordinates in bin *j* for N permutations:

We then compare the test statistic of the semivariogram from our final model, *t*, with the calculated values from the permutated locations (Figure S6). The *p*-value of the test is


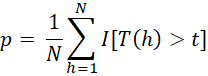


Where I[*a*>*b*]=1 if *a*>*b* and 0 otherwise. From this, we calculate the p-value to be 0.002 (none of the random statistics were greater than the empirical statistic), visualized in Figure S6. Therefore, there appears to be strong evidence of spatial dependence in PC 2 of the multidimensional scale.

**Figure S6. Histogram of randomly permuted test statistics, with the calculated value in red.**

# **Geostatistical modeling process**

We first utilized an intercept-only linear model with a spatial random effect:


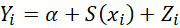


S(*x*) is a spatial random effect with covariance parameters σ^2^, φ, and τ^2^, estimated from the data, with shape parameter of the Matern function κ = 1.5 fixed, after evaluating the log-likelihoods of the final model at κ values at 0.5, 1, 1.5 and 2.

We can extend the model to include river catchment:


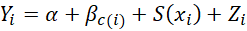


Where *c(i)* is the catchment associated with location *x_i_* for each location *i*, and
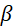
_1_=0. Parameter estimates for both models are summarized in Table S1. Catchment effects are relative to catchment 1.

Predicted genetic score across the city boundaries are shown in Figure S7 B, with the contributions from the covariate (Figure S7 A) and spatial random effects (Figure S7 C) separated. Catchment effects are relative to catchment 1.

**Table S1. Covariance parameters and coefficient estimates from geostatistical model.**

|  | **Intercept-only model** | | | **Intercept + nearest river** | | | |  |
| --- | --- | --- | --- | --- | --- | --- | --- | --- |
| **Parameter** | Estimate | Standard error | P value | | Estimate | Standard error | P value | |
| sigma^2^ | 4.75 | 1.107 | - | | 4.116 | 1.106 | - | |
| phi | 50.49 | 1.175 | - | | 40.496 | 1.119 | - | |
| tau^2^ | 0.185 | 1.857 | - | | 0.165 | 1.859 | - | |
| intercept | 0.066 | 0.161 | 0.683 | | 0.091 | 0.34 | 0.79 | |
| Catchment 2 | - | - | - | | -1.33 | 0.63 | 0.04 | |
| Catchment 3 | - | - | - | | 1.21 | 0.92 | 0.19 | |
| Catchment 4 | - | - | - | | 0.22 | 0.52 | 0.68 | |
| Catchment 5 | - | - | - | | 0.40 | 0.81 | 0.62 | |
| Catchment 6 | - | - | - | | 0.26 | 0.51 | 0.61 | |
| Catchment 7 | - | - | - | | 0.99 | 0.75 | 0.19 | |
| Catchment 8 | - | - | - | | -1.18 | 0.48 | 0.01 | |
| Catchment 9 | - | - | - | | 0.72 | 0.59 | 0.22 | |
| Catchment 10 | - | - | - | | 0.55 | 0.57 | 0.33 | |

**
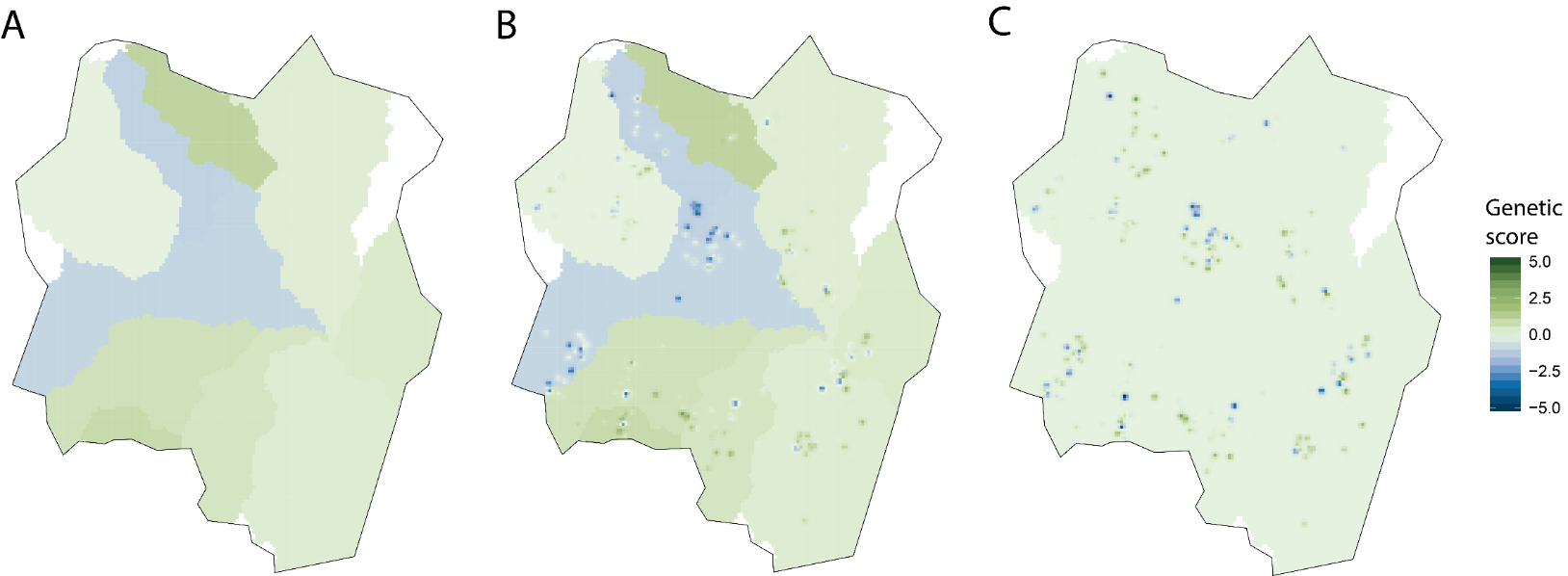
**

**Figure S7. Predictions from the intercept + river catchment model, A. Genetic score attributed to river catchment B. Total genetic score predictions across the city C. Estimated contribution of the spatial random effect**

# **Sensitivity analyses with household water source location**

Individuals tended to live near their water source locations, with a median distance of 65 meters (IQR 26-112). Therefore, we do not expect using water source location instead of household location to change the predictive ability of the river catchment variable, which exists on a much larger spatial scale >1km.

Regardless, we conducted a sensitivity analysis to compare results when using a geostatistical model using water source coordinates instead of household location. Although the small-scale spatial correlation changes, river catchment still significantly improves the fit of the model to the spatial-genomic patterns seen, although less significantly (LL -313.39 vs. -304.58, D= 17.623, p = 0.040). Coefficients for river catchments 2 and 8 remain distinct from the other catchments (Table S2).

**Table S2. Covariance parameters and coefficient estimates from geostatistical model using GPS coordinates of water source instead of household**

| **Parameter** | **Estimate** | **Standard error** | **P value** |
| --- | --- | --- | --- |
| sigma^2^ | 4.43 | 1.098 | - |
| phi | 22.46 | 1.330 | - |
| tau^2^ | 0.048 | 3.374 | - |
| intercept | 0.21 | 0.32 | 0.52 |
| Catchment 2 | -1.41 | 0.60 | 0.02 |
| Catchment 3 | 0.55 | 1.00 | 0.58 |
| Catchment 4 | 0.20 | 0.50 | 0.69 |
| Catchment 5 | -0.34 | 0.76 | 0.65 |
| Catchment 6 | 0.32 | 0.50 | 0.53 |
| Catchment 7 | 0.52 | 0.71 | 0.47 |
| Catchment 8 | -1.32 | 0.47 | 0.01 |
| Catchment 9 | 0.21 | 0.55 | 0.71 |
| Catchment 10 | 0.39 | 0.57 | 0.49 |

1. Diggle PJ, Tawn JA, Moyeed RA. Model-based geostatistics. J R Stat Soc Ser C (Applied Stat **2002**; 47:299–350. Available at: http://doi.wiley.com/10.1111/1467-9876.00113.
