# Supplementary material for: Spatial and Genomic Data to Characterize Endemic Typhoid Transmission
Source: Clin Infect Dis. 2021 Aug 31;74(11):1993–2000. doi: 10.1093/cid/ciab745 (PMC9187325; doi:10.1093/cid/ciab745)
Supplement: ciab745_suppl_Supplementary_Table_S1 [file ciab745_suppl_supplementary_table_s1.docx]

| **Characteristic** | **Value, n (%)** |
| --- | --- |
| **Clinical syndrome at presentation** |  |
| Non-focal sepsis | 140 (43) |
| Abdominal pain and/ or diarrhea and vomiting | 147 (45) |
| Encephalopathy | 11 (3) |
| Focal chest signs or abnormal radiograph | 7 (2) |
| Muskuloskeletal symptoms | 3 (1) |
| Febrile convulsion | 2 (0.6) |
| Meningism | 0 (0) |
| **Complication** |  |
| Severe anemia | 29 (8.9) |
| Hepatitis or cholecystitis | 13 (4) |
| Severe sepsis | 13 (4) |
| Gastrointestinal bleed | 13 (4) |
| Shock | 9 (3) |
| Intestinal perforation | 6 (2) |
| Severe malnutrition | 5 (2) |
| Meningitis | 1 (0.3) |

**Supplementary Table 1. Clinical syndromes and complications of 326 inpatients.**
